# Impact of Alkali and Silane Treatment on Hemp/PLA Composites’ Performance: From Micro to Macro Scale

**DOI:** 10.3390/polym13060851

**Published:** 2021-03-10

**Authors:** Percy Festus Alao, Laetitia Marrot, Michael David Burnard, Gregor Lavrič, Mart Saarna, Jaan Kers

**Affiliations:** 1 Department of Material and Environmental Technology, Tallinn University of Technology, Ehitajate tee 5, 19086 Tallinn, Estonia; jaan.kers@taltech.ee; 2 InnoRenew CoE, Livade 6, 6310 Izola, Slovenia; laetitia.marrot@innorenew.eu (L.M.); mike.burnard@innorenew.eu (M.D.B.); 3 Andrej Marušič Institute, University of Primorska, Muzejski trg 2, 6000 Koper, Slovenia; 4 Pulp and Paper Institute, Bogišićeva 8, 1000 Ljubljana, Slovenia; gregor.lavric@icp-lj.si; 5 Department of Mechanical and Industrial Engineering, Tallinn University of Technology, Ehitajate tee 5, 19086 Tallinn, Estonia; mart.saarna@taltech.ee

**Keywords:** hemp fibers, polylactic acid, biocomposite materials, mechanical properties, surface treatments

## Abstract

This study investigated the effect of hemp fiber pretreatments (water and sodium hydroxide) combined with silane treatment, first on the fiber properties (microscale) and then on polylactide (PLA) composite properties (macroscale). At the microscale, Fourier transform infrared, thermogravimetric analysis, and scanning electron microscopy investigations highlighted structural alterations in the fibers, with the removal of targeted components and rearrangement in the cell wall. These structural changes influenced unitary fiber properties. At the macroscale, both pretreatments increased the composites’ tensile properties, despite their negative impact on fiber performance. Additionally, silane treatment improved composite performance thanks to higher performance of the fibers themselves and improved fiber compatibility with the PLA matrix brought on by the silane couplings. PLA composites reinforced by 30 wt.% alkali and silane treated hemp fibers exhibited the highest tensile strength (62 MPa), flexural strength (113 MPa), and Young’s modulus (7.6 GPa). Overall, the paper demonstrates the applicability of locally grown, frost-retted hemp fibers for the development of bio-based composites with low density (1.13 to 1.23 g cm^−3^).

## 1. Introduction

The development of structural plant fiber composite components started about 80 years ago [1] and there is considerable interest in them nowadays because of the growing environmental and ecological pressures facing industries. Characteristics and properties of biocomposites have evolved, but improvements are still needed for the effective and durable use of plant fiber reinforcement of composites. While flax has been extensively studied in this aim [1], hemp fibers also show interesting specific properties for the reinforcement of composites [2]. The rougher surface of hemp fibers compared with flax fibers could positively influence the fiber/matrix adhesion [3]. Moreover, an increase in fiber surface roughness was found to facilitate the capillary flow in fibrous porous media, which suggests a smooth composite production process [4]. Adhesion between natural fibers and thermoplastic matrices is always questioned due to the hydrophilic character of natural fibers, which induces a decrease in the interfacial contact between the natural fiber and the matrix polymer, leads to dimensional changes and lower mechanical performance from possible moisture uptake in the composite and provides favorable conditions for the development of microorganisms. In the literature, various treatments have been investigated to improve adhesion between natural fibers and matrices, either by cleaning impurities from the surface of the fibers or by creating chemical bonding between the two components with coupling agent agents [5]. Bourmaud et al. [6] used a soft water treatment to clean flax fibers. Alkali treatments are used to remove hemicelluloses from natural fibers and can also partially remove other non-cellulosic components like lignin and pectin [7]. The removal of hemicellulose and lignin cover materials exposes more cellulose hydroxyl groups to alkali, which reduces the hydrophilic nature of natural fibers by replacing cellulose hydroxyl groups with less hydrophilic O−Na^+^ groups (Fiber-H + NaOH-> Fiber-O^−^ Na^+^ + H_2_O) and improves adhesion between fibers and matrix binders [5]. Silane coupling agents are used to improve the wettability of fibers by matrix polymers through graft copolymerization. Stable covalent bonds are eventually formed between alkoxy silanes and hydroxyl groups on the fiber surface [5,8].

Fiber treatments are often conducted to improve matrix adhesion within a composite, but they may degrade the fibers’ mechanical performance at the same time [9]. As highlighted by Liu et al. [5], the effect of treatment on the mechanical properties of the fibers and resulting composites should be understood to provide effective and profitable treatments. Moreover, Merotte et al. [3] showed that the improvement of interfacial shear strength brought on by a coupling agent (MAPP vs. PP) had much less influence on the mechanical properties of the composite than the nature of the fibers (flax or hemp) and their individualization. With low interfacial shear strength systems, such as plant fibers associated with polyolefin matrices like polypropylene, polyethylene, or polylactic acid (PLA), macroscale mechanical properties are governed by the fibers’ mechanical properties and bonding area rather than by interfacial bond strength [3]. A composite’s properties depend on multiple fiber parameters (mechanical performance, content, aspect ratio, orientation, individualization and dispersion [10], adhesion with matrix [11], and porosities), so it is difficult to unquestionably attribute an improvement or diminution of the composite mechanical properties to fiber treatment.

**Figure 1:**
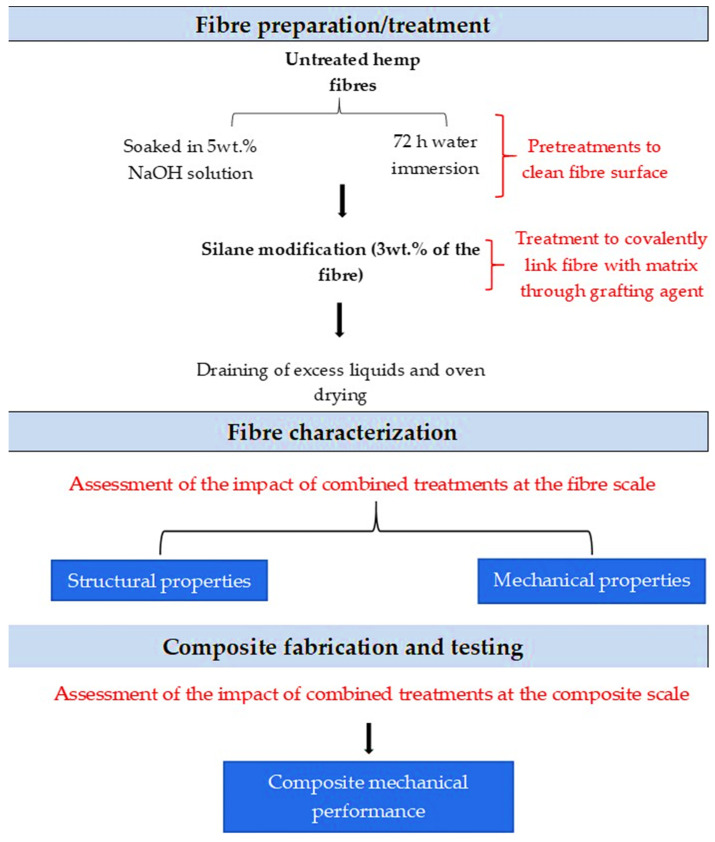
Schematic description of the current research aim and procedure.

This study investigates step by step the effect of different surface pretreatments (water and sodium hydroxide) combined with a silane treatment of an Estonian cultivated and frost retted hemp fiber, first on the single fiber microscale properties and then on the macroscale mechanical performance of their composites with a PLA matrix. This work aims to:

i. Show an accurate inference of the fiber pretreatments and treatment on the composite’s mechanical performance by linking the effects reported at the microscale, specifically the impact of the treatments on the fibers’ tensile properties, to their overall impact at the macroscale. The dual scales of observation bring a complementarity to the analysis, which is not often reported in the literature. Fiber composition, tensile mechanical properties, individualization, and dispersion are thoroughly studied, while the fiber content, aspect ratio, and orientation are carefully maintained as equivalent for all the composite formulations.
ii. Provide first-hand information on the suitability of Estonian hemp fibers for composite reinforcement. Indeed, these fibers are a by-product of cannabidiol production for medicinal applications and are currently considered as a waste. PLA is selected for its renewable and compostable properties, along with its comparable performance to common petrochemically derived alternatives [12]. Figure 1 presents a schematic of the main idea and procedure of this study.

## 2. Materials and Methods

### 2.1. Materials

Hemp fibers (Cannabis sativa, Tisza, Hungary) grown in Saaremaa, Estonia were used. Their properties have been characterized and described in previous work [13]. The stems were industrially decorticated by a mechanical process. PLA fibers (IngeoTM 4043D) from NatureWorks (Minnetonka, MN, USA) were used for the research. The polymer was 60 mm long, with a round cross-section, a finesse of 6.7 dtex and a density of 1.24 g cm^−3^.

### 2.2. Fiber Surface Treatments

#### 2.2.1. Water Treatment

Fibers were dried in an oven at 80 °C until constant weight to remove excess moisture and then washed by soaking in distilled water for 72 h at 23 °C according to Bourmaud et al. [6].

#### 2.2.2. Alkali Treatment

Hemp fibers were treated with a solution of 5 wt.% sodium hydroxide (NaOH). (Sigma-Aldrich, Saint Louis, MO, USA) Sodium (Na) granules were used in preparing the NaOH solution. Hemp fibers were soaked in the solution at room temperature (23 °C) for 4 h. Fibers were then washed in tap water to remove residual alkali by measuring the wastewater’s pH until it was about 7. Finally, the fibers were oven-dried at 80 °C until constant weight.

#### 2.2.3. Silane Treatment

**Figure 2:**
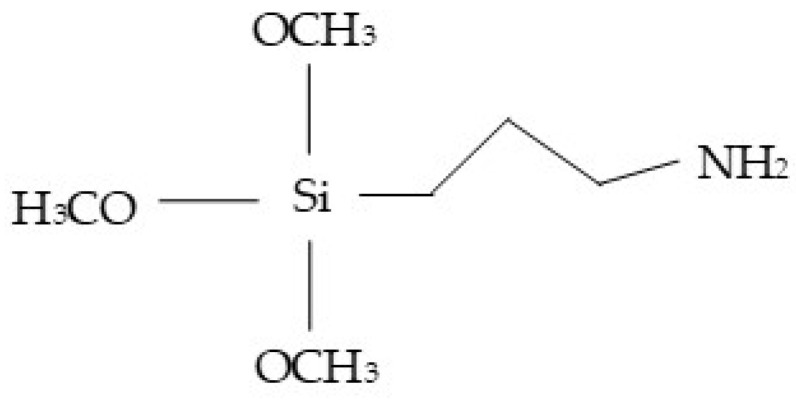
Structure of 3-Aminopropyl-triethoxy silane.

Hemp fibers were treated with an ethanol and water solution containing 3 wt.% silane coupling agent (3-Aminopropyl-triethoxy silane) whose structure is presented in Figure 2. The amount of silane was relative to the weight of hemp fibers. Silane was previously pre-hydrolyzed at room temperature for 2 h in an 80/20 vol % solution of ethanol/water. The pH of the solution was adjusted to 5 using acetic acid. Hemp fibers were soaked in the solution at room temperature for 2 h. Then, the hemp fibers were filtered and oven-dried at 80 °C until constant weight.

### 2.3. Fabrication of the Hemp Reinforced PLA (HPLA) Composites

Untreated (U_f_) and treated (distilled water (W_f_), water + silane (WS_f_), alkali (A_f_) and alkali + silane (AS_f_)) fibers were combined with PLA by compression moulding. Two composite types were produced from 30 wt.% (160.5 g of hemp fibers) + PLA and 50 wt.% (267.5 g of hemp fibers) + PLA in a metal frame (450 mm × 450 mm × 2 mm) using a hot press. Hemp and PLA fibers were mixed using a wide classic drum carder (300 mm batt width, 72 teeth per inch (tpi) and 100 g capacity). The mixture was dried in an oven for 4 h at 80 °C before compression at 180 °C and 3 MPa for 10 min. Neat PLA boards were also fabricated as a control. Abbreviations and descriptions for the composite boards are presented in Table 1.

### 2.4. Characterization of Hemp Fibers and HPLA Composites

#### 2.4.1. Chemical Composition by Fourier Transform Infrared (FTIR)

The spectroscopy was carried out to qualitatively identify the constituents of untreated and chemically treated hemp fibers and assess the effects of the treatments on the composition. Measurements were performed on a Nicolet™ iS50 FTIR Spectrometer (Thermo Fisher Scientific, Waltham, MA, USA) with ATR module from Thermo Scientific™. The fiber batches were conditioned in the spectrometer room for two weeks before analysis to ensure stable moisture content (MC). Analysis was performed on the fibers (not grounded) to preserve the internal organization of components. Bundles of hemp fibers were twisted by hand and placed on the ATR crystal. All FTIR spectra were collected with a spectrum resolution of 4 cm^−1^. A background scan of clean Zn–Se diamond crystal was processed before the sample scanning procedures. Ten replicates were tested for each batch with 22 scans per sample.

#### 2.4.2. Chemical Composition by Thermogravimetric Analysis (TGA)

Thermogravimetric analysis was performed on untreated and treated hemp fibers. The experiments were carried out using a NETZSCH STA 449F3 (NETZSCH-Gerätebau GmbH, Wittelsbacherstraße, Germany) in a nitrogen atmosphere (20 mL per min). For each sample (approximately 6 mg), three measurements were performed, beginning with an isothermal segment at 40 °C for 1 min, followed by dynamic heating from 40 °C to 600 °C at the rate of 2 °C min^−1^. Samples were held in an aluminum pan (Al_2_O_3_). Specimens were kept in the testing room at a relative humidity of 43 ± 10% and a temperature of 22 ± 1 °C for one week before the test.

#### 2.4.3. Microscopical Observations by Scanning Electron Microscopy (SEM)

SEM images of the fibers and composites were observed using a Zeiss Ultra 55 (FELMI-ZFE, Steyrergasse, Austria) at 20 kV, depth of 100nm and resolution of 50,000. For this observation, samples were carbon glued on an aluminum stub and then coated with an alloy of 2 nm thick gold (Au)/palladium (Pd) layer (80/20). For the composite samples, each specimen was mounted into Buehler EpoThin Epoxy glue before coating and observation.

### 2.5. Mechanical Properties of Unitary Hemp Fibers and Hemp/PLA Composites

Tensile tests were carried out on single hemp fibers for the five batches on a Zwick Roell Z010 (ZwickRoell GmbH & Co. KG, August-Nagel-Straße, Germany) tensile machine equipped with a 20 N measuring cell (Class 0.5, ISO 7500-1) at a speed of 1 mm per min. The gauge length was taken at 10 mm. For each batch, at least 50 fibers were tested. The mean diameter of each fiber was measured before testing (average of 3 points).

Tensile and flexural tests were performed on the composite samples in accordance with EVS-EN ISO 527 (Type 2) and EVS-EN ISO 14,125 (Class II) standard tests, respectively, using an Instron 8516 (Norwood, MS, USA) machine equipped with a load cell of 10 kN. The test was done at (43 ± 10)% RH, (22 ± 1) °C and test speed of 2 mm per min. Five (5) replicates per batch were used to evaluate the result, though four replicas were used for tensile strength of the UH composites due to sampling issues during testing. Specimen dimensions for the flexural test were 80 × 15 mm and thickness varied from 2–4 mm, while tensile test specimens had a dimension of 250 mm × 25 mm. An Extensometer L_o_ = 50 mm (model 2630-112, s/n 937) was attached to the test specimens to determine the elongation before failure. In addition, composite density was determined in accordance with EVS-EN ISO 1183-1 from five replicas, using a Mettler Toledo AX balance. Test pieces were 15 mm × 20 mm and conditioned following ISO 291.

### 2.6. Statistical Analysis

Statistical analysis and figures were done in R v4.0.2 (Vienna, Austria) [14] and RStudio v1.3.1073 (Boston, MA, USA) [15] using the tidyverse package [16] for data manipulation and plotting and the emmeans package [17] to compute and extract pairwise comparisons between treatments. The boot package was used to extract bootstrapped estimates and their bias-corrected accelerated (BCA) confidence intervals.

#### 2.6.1. Statistical Analysis of Fiber Properties

Separate linear models were fit to each natural log-transformed response (Modulus, Strain and Strength) since the raw response data violated the equal variance assumption for a linear model. Models were fit with pretreatment-treatment interactions and pretreatment conditions only. Interaction models were fit without the raw pretreatment condition (degrees of freedom = 206) and resulted in conditional pairwise comparisons between treatment effects (silane or none) in each pretreatment condition (alkali, water). Pretreatment-only models excluded samples with treatments (degrees of freedom = 143) and resulted in pairwise comparisons between each of the pretreatment conditions (raw, water or alkali). Extracted pairwise comparisons between each treatment were back-transformed to the original response scale and reported as the ratio between medians of the compared treatments. *p*-values for pretreatment comparisons were adjusted using Tukey’s method for a family of 3 estimates. Significance level for all *p*-values was set to 0.05.

#### 2.6.2. Statistical Analysis of Composite Tensile and Flexural Properties

Linear models were fit for each measured property (flexure strength, flexure modulus, tensile strength and tensile modulus). Both flexure (strength and modulus) and tensile modulus model had 7 and 42 degrees of freedom, while the tensile modulus had 7 and 41 degrees of freedom (due to some testing errors with a specimen). In each case, the response was log-transformed because the linear model’s equal variance assumption was violated. Each model was fit to pretreatment and treatment main effects as well as interaction effects between the pretreatment/fiber loading and treatment/fiber loading. Due to sample sizes, linear models and resulting pairwise comparisons were bootstrapped and their BCA confidence intervals calculated. The resulting estimates were the ratios between medians of compared treatments at specified fiber loading levels (30% or 50%) on the original scale. Reported *p*-values were based on the model values (not the bootstrapped values) and adjusted for a family of 6 comparisons using Tukey’s method. The significance level for all *p*-values was set to 0.05.

## 3. Results and Discussion

### 3.1. Chemical Composition by FTIR Analysis

**Figure 3:**
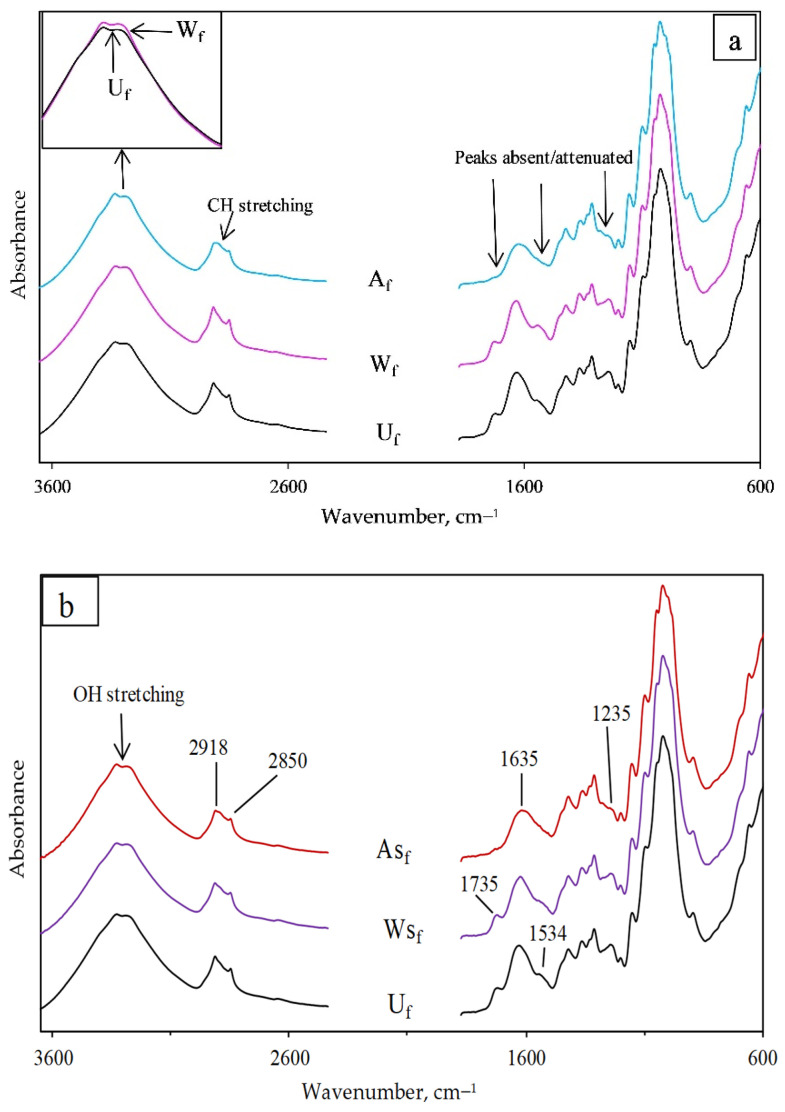
Vertically shifted FTIR spectra for (**a**) untreated (U_f_), distilled water (W_f_), and alkali-treated (A_f_) hemp fibers; (**b**) U_f_, water + silane (WS_f_) and alkali + silane (AS_f_) treated hemp fibers with the wavenumbers for differences observed.

For easier visualization, the average vertically shifted FTIR spectra are separated into two. Figure 3a displays the FTIR spectra for U_f_, W_f_, and A_f_, while Figure 3b shows the spectra for U_f_, WS_f_, and AS_f_. Qualitatively, U_f_ and W_f_ spectra appear similar; however, there is a higher absorbance in 3000–3600 cm^−1^ for W_f_ compared to U_f_. The 3000–3600 cm^−1^ corresponds to OH stretching vibrations, an increase of which depicts more OH functionality and lower hydrophilic properties [18]. This implies that some non-cellulosic polysaccharide was removed from the fiber surface, as shown by Bourmaud et al. [6]. Conversely, the A_f_ spectrum presents peak absence/reduction at about 1735 cm^−1^ and 1235 cm^−1^. The peak around 1735 cm^−1^ corresponds to C=O stretching vibration of conjugated carboxylic ester groups [19] of hemicellulose or wax [18], and the peak around 1235 cm^−1^ corresponds to C–O stretching of lignin acetyl groups [18,20]. Furthermore, the peak at 1635 cm^−1^ that shows C=O stretching in conjugated carbonyl of lignin or absorbed water is broader and attenuated [21]. Additionally, oscillations at 2918 cm^−1^ that correspond to C–H stretching in lignin’s aromatic hydrocarbon, methoxyl, and methylene groups [9], as well as oscillations at 2850 cm^−1^ related to symmetric C–H stretching of non-aromatic compounds present in the cellulose and hemicellulose components [20], are also reduced. Higher absorbance with better peak definition from 3000–3600 cm^−1^ is achieved by alkali-treated fibers.

**Figure 4:**
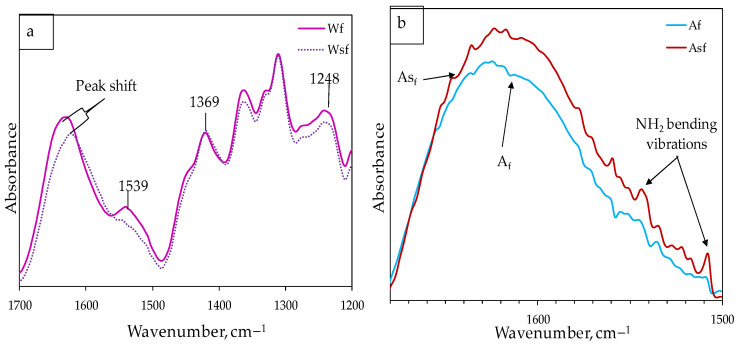
FTIR spectra for (**a**) degradation of a portion of lignin in WS_f_; (**b**) silane coupling effect after treatment of alkali modified fibers.

Ostensibly, it appears that the spectrum of WS_f_ and W_f_ is also similar, but Figure 4a clearly shows that the additional silane treatment induces a peak shift from 1635 cm^−1^ to 1624 cm^−1^ and attenuation at about 1539 cm^−1^, 1369 cm^−1^, and 1248 cm^−1^. Similar peak shifts have been reported in past research after silane pretreatments of hemp fibers [9,18,21]. This suggests that the ensuing silane treatment led to the extraction of some hemicellulose, wax and lignin fiber contents. In Figure 4b, it was also discovered that the subsequent silane treatment causes higher peak intensity with new peaks that could be related to NH_2_ bending vibrations in amino silane between 1500–1680 cm^−1^, also reported by Panaitescu et al. [21]. We can infer from our results that the water treatment did not affect the lignin content of hemp fibers, but it slightly increased the functional OH due to the removal of some water-soluble polysaccharides. Alkali treatment was effective in extracting pectins, hemicellulose, and lignin content, and silane treatment showed slight removal of intercellular content, especially for water pretreated hemp fibers with some new peaks that could be due to silane molecule coatings on the fiber surface following water/alkali pretreatments.

### 3.2. Chemical Composition by Thermogravimetric Analysis (TGA)

**Figure 5:**
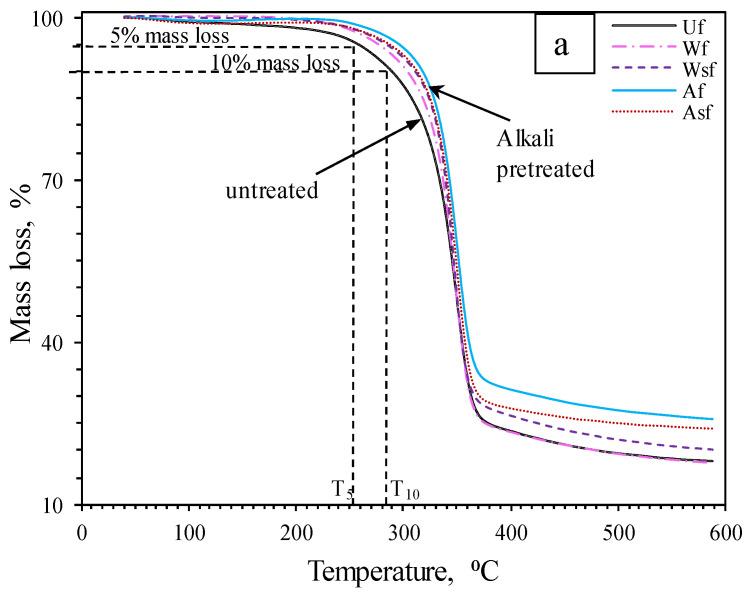

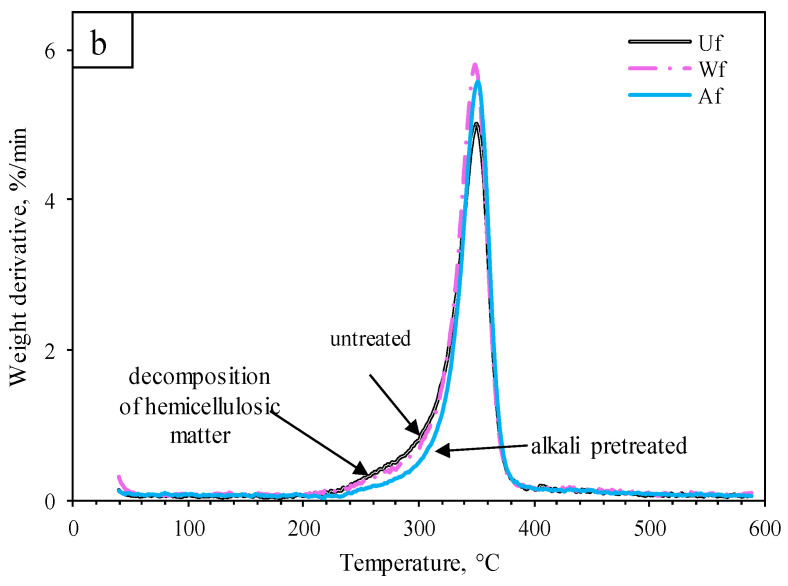
(**a**) TGA curves for U_f_, W_f_, WS_f_, A_f_, AS_f_; (**b**) dTGA curve for U_f_, W_f_ and A_f_ batches.

TGA curves for all batches (Figure 5a) display two main weight losses, while for clarity, only the differential thermogravimetric analysis (dTGA) (Figure 5b) for untreated, water- and alkali-treated fibers is presented. In Figure 5a, the observed mass loss between 39–160 °C was attributed to the evaporation of water from the fibers. The estimated mass loss in this temperature range is shown in Table 2. A loss of approximately 1.4% was obtained for U_f_ compared to 0.3% for the batches of treated fibers. The lower moisture content observed for W_f_ and A_f_ compared to U_f_ is in accordance with the FTIR results for which pretreated fibers showed decreased hydrophilicity associated with the removal of non-cellulosic polysaccharides. Other studies [18,20] reported a similar decrease of mass loss in the 39–160 °C region for fibers that underwent alkali treatments.

The second degradation appears between 160 and 600 °C with a peak of around 346 °C. Placet et al. [22] highlighted the superimposition of several degradations in this range of temperatures. The majority of hemicellulose matter degrades between 180 and 280 °C, while the remaining hemicelluloses and formed by-products decompose from 280 to 600 °C. However, cellulose decomposes roughly between 325 and 400 °C, and lignin decomposes between 150 and 450 °C. Here, the second degradation corresponds then to the decomposition of amorphous polysaccharides (hemicelluloses and pectin) taking place between 180 and 280 °C, where a shoulder is visible on the dTGA curve, in addition to the decomposition of cellulose and lignin constituents and formed by-products. The remaining mass corresponds to the ash or non-polysaccharide contents [6]. The composition of the studied hemp fibers was investigated in previous work [13] and showed cellulose (77.4%), hemicellulose (8.3%), solubles (12.6%), and lignin (1.4%). To further investigate the fiber treatment’s effectiveness, temperatures corresponding to a 5% weight loss (T_5_) and a 10% weight loss (T_10_) were considered (Table 2). It can be seen that after water and alkali pretreatment, the values shifted to higher temperatures (i.e., from 254 °C and 289 °C for U_f_ to 272 °C and 299 °C for W_f_ and 292 °C and 313 °C for A_f_). This translates to the removal of hemicelluloses and pectins, which were included in the solubles content reported above.

The reduced truncation of A_f_ dTGA curve (Figure 5b) compared to U_f_ also confirms the removal of hemicellulose matter [6], which corresponds to the FTIR observation. Likewise, Table 2 reveals an improvement in thermal stability for WS_f_ compared to W_f_, which is mainly due to the effect of silane molecules coating the fiber surface [6]; though, there seems to also have been possible additional extraction of some non-cellulosic fiber components during silane treatment as indicated by the FTIR results. Such non-cellulosic components removal after silane treatment has also been highlighted by Panaitescu et al. [21], and a similar rise in thermal degradation temperatures was obtained for fibers treated with silane after washing in water by Dayo et al. [18]. The observed T_5_ and T_10_ values for WS_f_, A_f_, and AS_f_ are comparable to values reported in the literature [18,20].

### 3.3. Scanning Electron Microscopical (SEM) Observations

**Figure 6:**
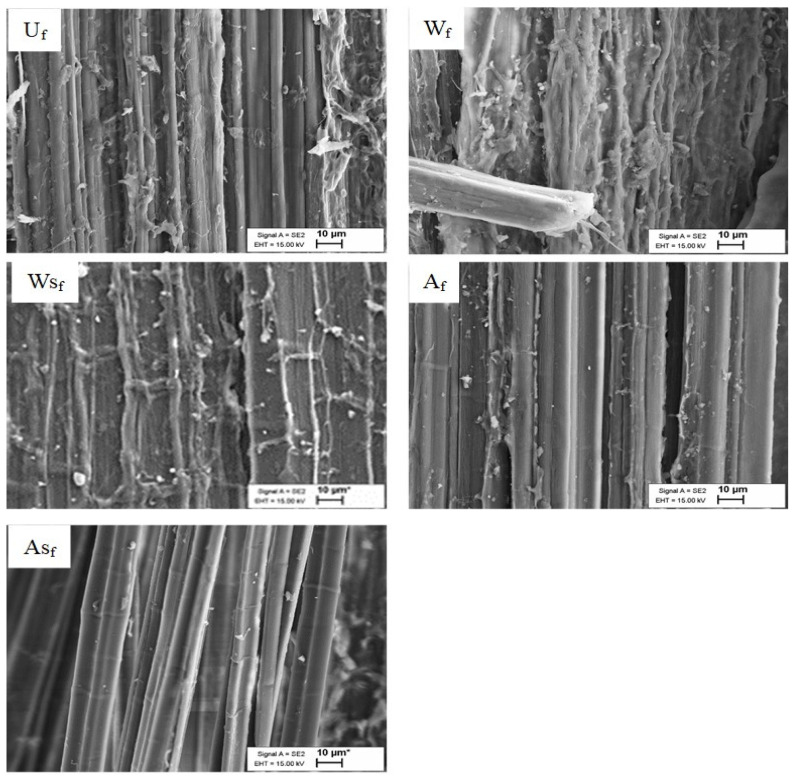
SEM images of (U_f_) untreated, (W_f_) water-treated, (WS_f_) water + 3% silane-treated, (A_f_) 5% alkali-treated, and (AS_f_) 5% alkali + 3% silane-treated hemp fiber surfaces.

**Figure 7:**
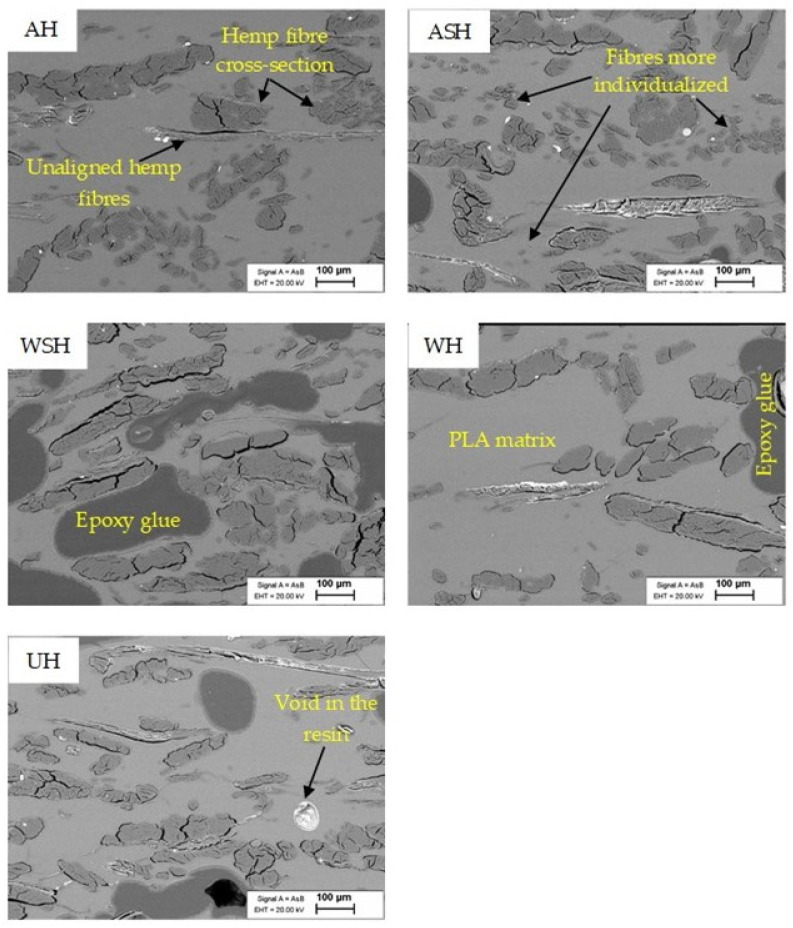
Cross-section SEM images of UH, WH, WSH, AH, and ASH composites from 30 wt.% hemp fibers.

Observation of the SEM images presented in Figure 6 shows a cleaner and clearer surface for the A_f_- and AS_f_-treated fibers. This cleaner appearance is associated with substantial removal of hemicellulose and lignin from the fiber surface [5,9,23,24]. However, compared to the untreated fibers, W_f_ does not appear to have removed non-cellulosic contents (most likely wax and lignin contents), while the fibers subsequently treated with silane (WS_f_ and AS_f_) show a slightly smoother surface compared to those of W_f_ and A_f_, respectively. This outcome could be due to the additional removal of pectin and hemicellulose by the ethanol/water mixture and the formation of a siloxane layer on the fiber surface due to condensation of the silane groups that are reported to be more visible with higher amounts of silane modification (5–20%) [8]. These generally agree with the FTIR and TGA analyses and show the differences in surface structure for the hemp fibers after treatments. SEM micrographs of the HPLA composites (Figure 7) visibly prove that the fiber treatments improved fiber distribution and individualization within the matrix in the highest order from ASH, AH, and WSH to WH and UH as a result of the non-cellulosic content removal, which has also been reported in past studies [25,26]. Commonly, all the composites show good fiber alignment.

### 3.4. Mechanical Properties

#### 3.4.1. Tensile Properties of the Hemp Fibers

**Figure 8:**
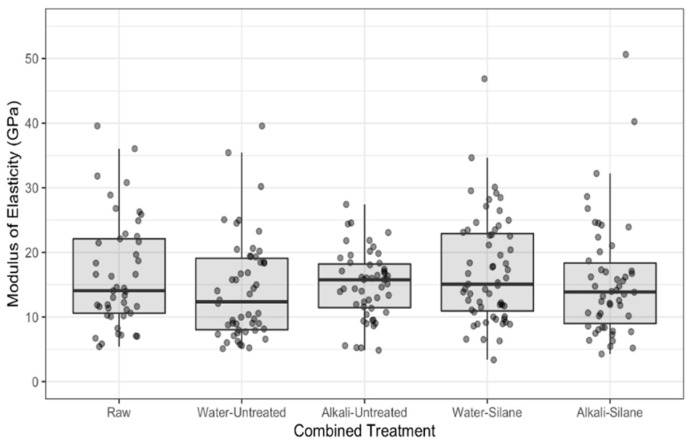
Modulus of elasticity for raw hemp fibers and fibers with combined treatments.

**Figure 9:**
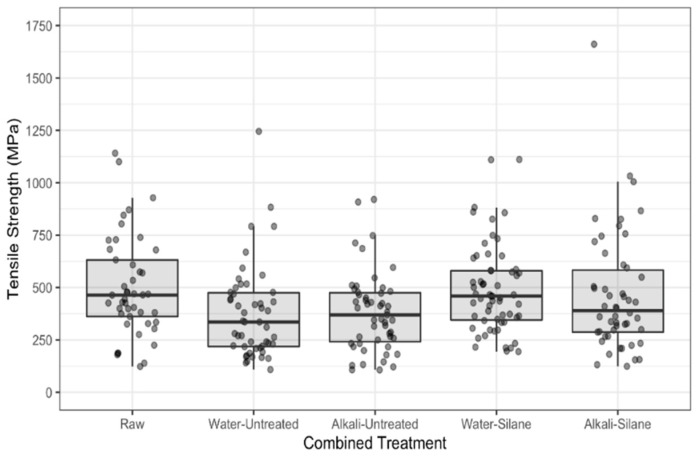
Tensile strength of raw hemp fibers and fibers with combined treatments.

**Figure 10:**
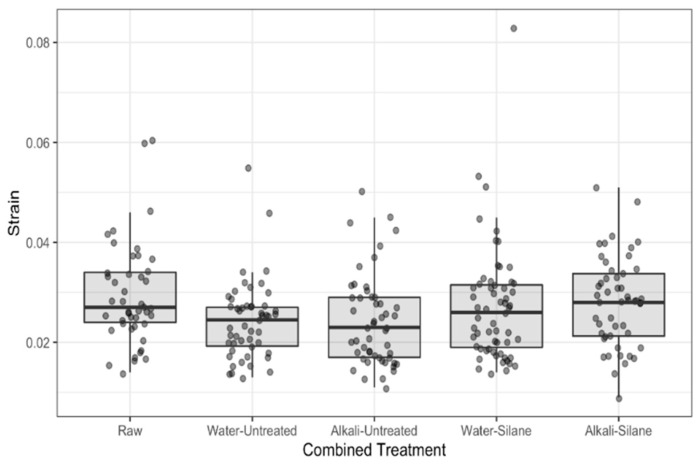
Strain of raw hemp fibers and fibers with combined treatments.

Mechanical properties (modulus of elasticity (MoE), strength and strain) are presented in Figure 8, Figure 9 and Figure 10, respectively. On the figures, the lower and upper hinges correspond to the first and third quartiles (the 25th and 75th percentiles). We see widely scattered values for all properties (Figure 8, Figure 9 and Figure 10, Table 3), as is often reported in the literature for hemp and other plant fibers [2,26].

Table 4 presents the effect of pretreatments (none = raw fibers, water, and alkali) on the tensile properties of hemp fibers as a ratio between medians of the given treatments. We note that MoE of the hemp fibers was not affected by water or alkali pretreatments (confidence intervals for the ratios include 1). However, the tensile strength and elongation at break were both reduced after water and alkali pretreatments. The median tensile strength of raw fibers was 1.36 times greater than water pretreated fibers (95% CI: 1.06 to 1.76) and was 1.33 times greater than fibers pretreated with alkali (95% CI: 1.03 to 1.72). Median elongation at break was 1.23 times greater for raw fibers than alkali pretreated fibers (95% CI: 1.04 to 1.44) and was 1.2 times greater for raw fibers than for water pretreated fibers (95% CI: 1.02 to 1.41). There was no meaningful difference in the tensile properties between fibers pretreated with water and alkali (95% CI’s include 1 in each case).

Water pretreatment generates a water uptake by the fibers, which occurs under two states in natural fibers, depending on the moisture content [22,27,28]: (i) Water bound to the different biopolymers constituting the cell walls and middle lamella, involving the formation of hydrogen bonds with hydroxyl groups OH, and (ii) free water that fills voids (micro- and macropores of cell walls and lumens) and is retained by capillary forces. Garat et al. [29] measured moisture content of 62.8 ± 0.7% for hemp fiber bundles in immersion (compared to 60.9 ± 0.7% for flax fiber bundles). Moreover, Pejic et al. [28] showed that lignin removal decreases the moisture sorption and increases the water retention ability of hemp fibers. Marrot et al. [13] highlighted that the raw hemp fibers of this study display a particularly low lignin content, which is also confirmed by the TGA analysis. We can then assume that our fibers show high water retention ability during water pretreatment. Pectins and hemicelluloses from the surface, cell wall and middle lamella are removed during water pretreatment, confirmed by the FTIR and TGA results, and we suspect changes in the component arrangements of the S2 layer that consists of highly crystallized cellulose microfibrils embedded in an amorphous polysaccharide matrix (pectins and hemicelluloses). A decrease of the median tensile strength by 36% after water pretreatments can be explained by a component rearrangement in the S2 layer, which controls the mechanical properties of the whole fiber. Le Duigou et al. [30] also observed an alteration of the structural cohesion (cell-wall peeling process) of a flax fiber following water treatment.

Regarding alkali pretreatment, the median tensile strength was found to be 33% lower than untreated fibers. The effects of alkaline treatments on the mechanical properties of hemp fibers are controversial in the literature; Kabir et al. [9], Väisänen et al. [23] and Islam et al. [31] observed a deterioration of tensile properties (strength and modulus) that they attributed to lignin removal and other non-cellulosic components that reinforce the fibers and to potential degradation of cellulose chains [32]. On the contrary, Sawpan et al. [24] and Sair et al. [33] observed an increase of tensile properties for hemp fibers after alkali treatments that they attributed to a relaxation and reorganization of the microfibrils along the principal axis of the fiber, resulting in a more rigid structure thanks to the elimination of lignin and hemicellulose components. Besides a reduction of lignin and hemicellulose amounts [29,30], authors report a transformation of cellulose II to cellulose I [33] and an augmentation of the cellulose crystallinity [31] after application of an alkaline pretreatment to the fibers.

Furthermore, the effect of silane treatment on the tensile properties of hemp fibers pretreated with water and alkali is also shown in Table 4. In the case of alkali pretreatment, silane did not affect MoE, but fiber elongation and tensile strength were greater (strain: 1.18 times, 95% CI: 1.03 to 1.35; tensile strength: 1.19 times, 95% CI: 0.97 to 1.45). When pretreated with water, both MoE and tensile strength were greater when treated additionally with silane (MoE: 1.38 times, 95% CI: 1.13 to 1.67; tensile strength: 1.23 times, 95% CI: 1.02 to 1.49). There was a moderate increase in fiber elongation in this case as well. Silane couplings with cellulose microfibrils formed a layer of chemicals on the fiber surface, acting like a coating, which agrees with the FTIR and TGA results. During the tensile test, an additional shear resistance was brought by the layer of chemicals that attached to the microfibrils. Shear resistance creates higher elongation of the microfibrils, resulting in increased deformation of the fiber as highlighted by Kabir et al. [9].

#### 3.4.2. Tensile Properties of HPLA Composites

**Figure 11:**
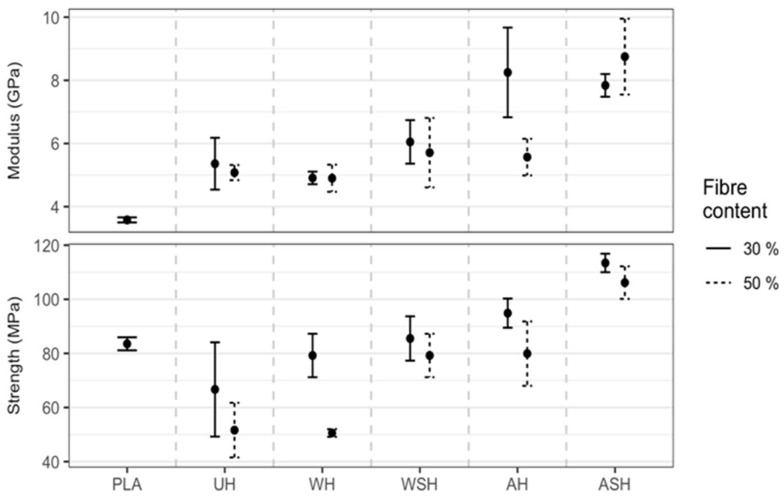
Medians (●) for YM and TS at 30 and 50 wt.% hemp fiber content for untreated (UH) and treated (WH, WSH, AH, and ASH) compared to neat PLA (bars show the one interquartile range on either side of the median).

From the TS results presented in Figure 11, UH shows a lower outcome of 48 ± 2.4 MPa (30 wt.%) and 37 ± 7 MPa (50 wt.%), compared to the neat PLA, 51 ± 0.45 MPa. This indicates an ineffective reinforcement of PLA with untreated hemp fibers. There was a slight increase of about 6% for WH compared to UH (30 wt.%), but no meaningful improvement was achieved for WSH compared to WH. On the other hand, alkali pretreatment of the hemp fibers significantly boosted the composite TS by about 14%; subsequent treatment with silane added another 10% compared to AH. At the fiber level, both water and alkali pretreatments decreased TS of the fibers by about 30%. The increase in TS for WH and AH compared to the untreated hemp fiber reinforced PLA composites, despite the treatment’s negative impact on fiber performance, can be attributed to: (1) The better level of fiber individualization as observed by SEM technique, which induces a higher aspect ratio, and (2) the enhanced PLA-fiber bonding after removal of water-soluble polysaccharides in water pretreatment and removal of pectin, wax and intercellular components in alkali pretreatment. Removal of these components exposes more hydroxyl groups and increases access to cellulose sites for interlocking with the PLA matrix, which corroborates previous studies [23,34,35]. At the fiber scale, silane treatment increased the TS of hemp fibers pretreated with water and alkali. Additional improvement to TS observed on composites at the macroscale after silane treatment was attributable to higher fiber performance and improved fiber compatibility with the polymer matrix brought on by the silane couplings.

Regarding the 50 wt.% HPLA composites, there was generally a decrease in TS compared to 30 wt.%, which was ascribed to insufficient wetting of the fibers by the PLA matrix [24,26] and, consequently, an inefficient load transfer between fibers and matrix. Sawpan et al. [23] also showed that there was no linear improvement in TS with an increase in fiber loading from 10–30 wt.%; moreover, previous studies show that 30 wt.% was the maximum fiber volume fraction at which optimum composite mechanical performance was achieved [20,36]. From the result of the specimens’ Young’s modulus (YM), a significant rise of about 37% and 23.5% was obtained with reinforcements of 30 and 50 wt.% U_f_ compared to neat PLA due to much higher elastic modulus of the hemp fibers (Figure 8) compared to PLA (approx. 3.8 GPa based on technical data). At the fiber scale, hemp fibers’ MoE was not affected by water or alkali pretreatments and showed only a slight increase for WSf. However, on the composite scale, water pretreatment increased YM by 19 and 23% at 30 and 50 wt.%, respectively, while alkali treatment offered an even more superior outcome of 29 and 44%. Following the additional silane treatment of W_f_ (i.e., WSH), a further improvement of 6 and 15% was achieved at 30 and 50 wt.%, respectively. This mirrors the outcome at the fiber scale; though not meaningful at 30 wt.%, it was very significant at 50 wt.%, indicating better entrapment of the PLA by the fiber interpenetrating network that is due to silane molecules coating the fiber surface, as described by Xie et al. [37].

Likewise, for TS, YM declined at 50 wt.%, implying inadequate fiber wetting by the PLA matrix as earlier mentioned. We clearly observed that UH exhibited the most meaningful reduction (18%) in this regard, which is probably due to the increasingly less favorable matrix/fiber interface with higher fiber content. Overall, ASH (30 wt.%) exhibited the best outcome of 8.51 ± 0.2 GPa, representing a notable increase of 1.5 times (95% CI: 1.40 to 1.64) that of UH.

#### 3.4.3. Flexural Properties of HPLA Composites

**Figure 12:**
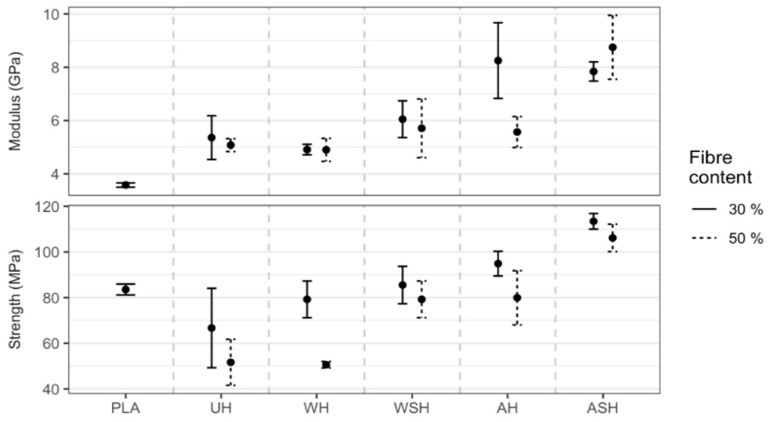
Medians (●) for FM and FS at 30 and 50 wt.% hemp fiber content for untreated (UH) and treated (WH, WSH, AH, and ASH) compared to neat PLA.

Median flexural strength (FS) and modulus (FM) for the specimens are presented in Figure 12. Neat PLA performed better in FS than UH, WH, and WSH composites, which gave lower outcomes. The reduction in FS compared to neat PLA, after reinforcement, was WSH < WH < UH at both 30 and 50 wt.%, implying improvement in the bonding between PLA and hemp fibers following water and a combination of water and silane fiber treatments. Alkali- and silane-treated hemp fiber reinforced composites (ASH) exhibited the most significant boost in FS (34% at 30 wt.% and 30% at 50 wt.%), compared to neat PLA, and was, significantly, 1.5 times (95% CI: 1.29 to 1.74) and 2 times (95% CI: 1.92 to 2.38) greater than 30 and 50 wt.% UH, respectively. AH also showed a meaningful 13% improvement at lower fiber content compared to neat PLA. As observed, there was a similar reduction in FS with an increase in the composite fiber content from 30 to 50 wt.%. The most notable reduction was by WH (33%), UH (26%) and AH (12%), while there was no meaningful decrease in performance for either ASH or WSH even though both exhibited about 3% lower FS at 50 wt.%. Compared to AH, there was an insignificant increase at 30 wt.% by ASH, but at 50 wt.%, there was a reasonably better outcome (1.4 times AH; 95% CI: 1.29 to 1.50), showing the positive influence of additional silane treatment on the hemp fibers. The higher outcomes for AH and ASH compared to UH was consistent with earlier presented results from FTIR, TGA, and SEM and also agrees with past studies [18,24,35].

Similar to YM, composite FM also increased by about 49% after the PLA was reinforced with untreated hemp fibers (30 wt.%) and, ultimately, by over 120% when treated hemp fibers (alkali + silane) were used. The outcome for UH compared to neat PLA was also inferred to be due to the superior elastic modulus of the hemp fibers, and the further consequential boost by ASH was the result of enhanced PLA/hemp fiber bonding after alkali and silane treatments. When we compared all composites, the increase in FM was UH = WH < WSH < AH < ASH. Generally, composite FM also decreased with an increase in fiber content, though we noticed a 6% rise for ASH at 50 wt.%, which was not significant and implied that there was no further improvement in performance.

Consistent with past studies, our research reported better composite flexural performance after alkali and silane fiber pretreatments [18,24,33,34], and the 8.58 GPa FM obtained in the current study for this particular treatment approach represents one of the best outcomes achieved using the hot press method and a fiber content of 50 wt.%. Hu and Lim [34] employed a similar fabrication and treatment method (i.e., hot pressing and alkali treatment, respectively), though with short fibers, and obtained slightly lower average FS results of 87.5 MPa for alkali-treated hemp fiber reinforced PLA composites at 30 wt.%. Sawpan et al. [24] obtained FS and FM of about 95 MPa and 6.59 GPa for alkali-treated, long-aligned 35 wt.% hemp fiber reinforced PLA composites that declined by about 29% and 15%, respectively, at 40 wt.%.

In addition to the mechanical properties discussed above, average density, specific tensile and flexural strength values for the HPLA composites are given in Table 5. Density of the neat PLA board is 1.24 g cm^−3^, which is the same value stipulated by the manufacturer and confirms the effectiveness of the fabrication approach. Densities for the composites are 1.13 to 1.23 g cm^−3^. There appears to be no significant difference in densities for composites of similar fiber content, but the 50 wt.% composites exhibit a slightly lower density compared to the 30 wt.% composites. Overall, the alkali pretreated and alkali + silane-treated hemp fiber reinforced composites show slightly higher density compared to the other composite variants, which could be due to consistency in hemp fiber content following the removal of most non-cellulosic elements. Lower density of the 50 wt.% composites could be the result of more voids, which decrease resin flow through the fibers at higher contents, and differences across all samples may also arise from fabrication irregularities, which may occur from the manual composite manufacturing processes. Pappu et al. [35] achieved specific TS and FS of (27.9 ± 2.5) σ/ρ and (64.9 ± 1.13) σ/ρ for neat PLA, which increased by 27% after incorporation of hybrid fibers of hemp and flax (composite density of 1.19 ± 0.02 g cm^−3^). Hu and Lim [34] obtained specific TS of 34.5 σ/ρ and 35.8 σ/ρ for untreated and alkali-treated hemp fiber reinforced PLA composites, respectively, at 50 wt.%. Compared to these past studies, the current study showed slightly higher outcomes. The use of such low-density composites could offer a suitable alternative to composites from synthetic fibers, reduce carbon dioxide emissions and increase energy savings in transportation applications.

## 4. Conclusions

This study was an in-depth investigation into the effect of different hemp fiber surface pretreatments (water and sodium hydroxide) combined with silane treatment. At the microscale, FTIR, TGA, and SEM investigations highlighted structural alterations in the fibers, with the removal of targeted components and rearrangement in the cell wall. These structural changes influenced unitary fiber properties. At the fiber microscale, preliminary treatment tended to reduce tensile strength and elongation at break but did not affect the modulus of elasticity. Silane treatment improved tensile strength for both pretreatments and modulus of elasticity after water pretreatment. At the macroscale, both pretreatments increased the composites’ tensile properties, despite their negative impact on fiber performance. This improvement was the result of a better level of fiber individualization after pretreatment and enhanced PLA-fiber bonding induced by the removal of water-soluble polysaccharides during water pretreatment and removal of pectin, wax and intercellular components during alkali pretreatment. Additionally, silane treatment improved composite performance thanks to the higher performance of the fibers themselves and improved fiber compatibility with the polymer matrix brought on by the silane couplings. This study showed successful development of low-density composites suitable for transportation applications, which will allow for a reduction of weight and carbon dioxide emissions. Future study will examine the influence of these chemical treatments on composite moisture/humidity sensitivity and fire performance.

## Figures and Tables

**Table 1:** Nomenclature used for polylactic acid (PLA), untreated and treated hemp fiber reinforced polylactide composites.

Abbreviation	Samples
Neat PLA	Unreinforced polylactic acid boards from 100% PLA fibers.
UH	Untreated hemp fiber (U_f_) reinforced polylactide composites.
WH	Water-treated hemp fiber (W_f_) reinforced polylactide composites.
WSH	Combined water- and silane- (WS_f_) treated hemp fiber reinforced polylactide composites.
AH	Alkali-treated hemp fiber (A_f_) reinforced polylactide composites.
ASH	Combined alkali- and silane- (AS_f_) treated hemp fiber reinforced polylactide composites.

**Table 2:** First TGA mass loss at 160 °C and temperatures corresponding to 5% and 10% weight loss for untreated and treated hemp fibers.

Sample	First Mass Loss (%) at 160 °C	T_5_ (°C)	T_10_ (°C)
U_f_	1.4	254	289
W_f_	0.3	272	299
WS_f_	0.3	278	305
A_f_	0.3	292	313
AS_f_	0.3	288	310

**Table 3:** Means and medians of tensile data for all combined treatments.

	Modulus (GPa)				Tensile Strength (MPa)				Strain%			
Combined	Mean	SD *	Median	IQR **	Mean	SD	Median	IQR	Mean	SD	Median	IQR
Raw	16.6	8.5	14.1	11.5	500	239	464	270	2.93	1.02	2.70	1.00
Water-Untreated	14.3	7.9	12.4	11.1	376	220	336	257	2.43	0.78	2.45	0.78
Alkali-Untreated	15.0	5.2	15.8	6.7	381	189	369	234	2.42	0.91	2.30	1.20
Water-Silane	17.2	8.3	15.1	12.0	490	210	459	236	2.71	1.16	2.60	1.25
Alkali-Silane	15.6	9.0	13.9	9.4	466	287	390	295	2.81	0.89	2.80	1.25

* SD = Standard Deviation; ** IQR = Interquartile Range.

**Table 4:** Effect of pretreatments (raw fibers, water and alkali) on the tensile properties of hemp fibers and comparison of tensile properties for hemp fibers before and after silane treatment (with water and alkali pretreatments).

	Tensile Strength			Modulus			Strain		
Comparison	Ratio	95% CI	*p*-Value	Ratio	95% CI	*p*-Value	Ratio	95% CI	*p*-Value
Raw/Alkali	1.3	1.03 to 1.72	0.0255 *	1.05	0.83 to 1.33	0.8647	1.23	1.04 to 1.44	0.0092 **
Raw/Water	1.36	1.06 to 1.76	0.0131 *	1.18	0.93 to 1.5	0.2130	1.20	1.02 to 1.41	0.0261 **
Alkali/Water	1.03	0.8 to 1.32	0.9641	1.12	0.89 to 1.41	0.4459	0.98	0.83 to 1.14	0.9289
Silane/Untreated|Alkali	1.19	0.97 to 1.45	0.0938	0.97	0.8 to 1.18	0.7777	1.18	1.03 to 1.35	0.0187 *
Silane/Untreated|Water	1.38	1.13 to 1.67	0.0013 **	1.23	1.02 to 1.49	0.0294 *	1.09	0.95 to 1.24	0.2041

* *p* < 0.05, ** *p* < 0.01. *p*-values were adjusted using Tukey’s method for a family of 3 estimates. Comparisons can be interpreted as the ratio between Silane and Untreated fibres that were pretreated with Alkali (Silane/Untreated|Alkali) or Water.

**Table 5:** Average density and specific mechanical properties of the neat PLA and HPLA composites.

	Specimen	Density (g/cm^−3^)	Specific TS σ/ρ	Specific FS σ/ρ		Density (g/cm^−3^)	Specific TS σ/ρ	Specific FS σ/ρ
100%	PLA	1.24 ± 0.01	41.29 ± 0.36	68.19 ± 2.03				
30 wt.%	UH	1.19 ± 0.11	40.19 ± 2.19	60.92 ± 10.51	50 wt.%	1.13 ± 0.05	33.06 ± 6.60	47.24 ± 5.68
	WH	1.20 ± 0.13	42.46 ± 6.05	67.05 ± 4.54		1.13 ± 0.10	42.85 ± 4.39	47.51 ± 5.97
	WSH	1.18 ± 0.05	43.94 ± 3.15	70.47 ± 4.01		1.13 ± 0.10	45.90 ± 3.29	71.63 ± 4.50
	AH	1.23 ± 0.02	45.20 ± 4.67	77.22 ± 2.61		1.16 ± 0.06	45.27 ± 1.98	72.59 ± 8.03
	ASH	1.21 ± 0.05	51.00 ± 2.64	93.64 ± 2.69		1.16 ± 0.03	46.67 ± 6.33	94.22 ± 6.68

## Data Availability

Not applicable.
