# Correction: Alao et al. Impact of Alkali and Silane Treatment on Hemp/PLA Composites’ Performance: From Micro to Macro Scale. *Polymers* 2021, *13*, 851

**DOI:** 10.3390/polym13162777

**Published:** 2021-08-18

**Authors:** Percy Festus Alao, Laetitia Marrot, Michael David Burnard, Gregor Lavrič, Mart Saarna, Jaan Kers

**Affiliations:** 1 Department of Material and Environmental Technology, Tallinn University of Technology, Ehitajate tee 5, 19086 Tallinn, Estonia; jaan.kers@taltech.ee; 2 InnoRenew CoE, Livade 6, 6310 Izola, Slovenia; laetitia.marrot@innorenew.eu (L.M.); mike.burnard@innorenew.eu (M.D.B.); 3 Andrej Marušič Institute, University of Primorska, Muzejski trg 2, 6000 Koper, Slovenia; 4 Pulp and Paper Institute, Bogišićeva 8, 1000 Ljubljana, Slovenia; gregor.lavric@icp-lj.si; 5 Department of Mechanical and Industrial Engineering, Tallinn University of Technology, Ehitajate tee 5, 19086 Tallinn, Estonia; mart.saarna@taltech.ee

The authors wish to make the following two corrections to this paper [1]: the graphical abstract on the website contains an image wrongfully labeled, and Figure 11 depicts the same data as Figure 12 in the original version of the published article. We apologize for the original errors. The graphical abstract and Figure 11 should be updated to correct this oversight.

**1.** In the graphical abstract, an image was mislabeled as “Tensile properties to be corrected”. The graphical abstract was replaced with the following image:


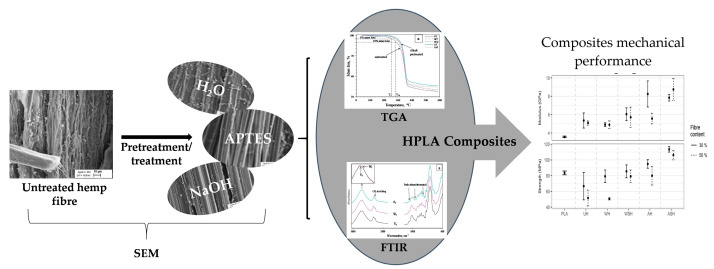


**2.** In Section 3.4.2. “Tensile properties of HPLA composites” of Results and Discussion on Page 14, Figure 11 was replaced with the updated data regarding the Medians (●) for YM and TS at 30 and 50 wt.% hemp fiber content for untreated (UH) and treated (WH, WSH, AH and ASH) compared to neat PLA (bars show the one interquartile range on either side of the median).

**Figure 11:**
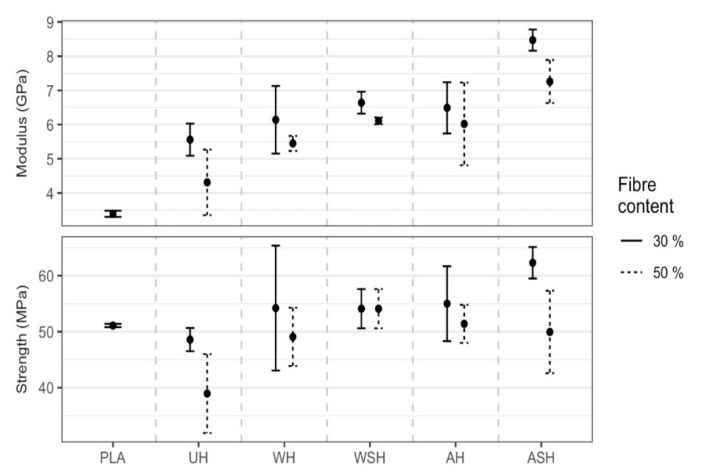
Medians (●) for YM and TS at 30 and 50 wt.% hemp fiber content for untreated (UH) and treated (WH, WSH, AH and ASH) compared to neat PLA (bars show the one interquartile range on either side of the median).

The authors apologize for any inconvenience caused to the readers by these changes. These changes have no material impact on the conclusions of our paper.
